# Interplay Between Traditional and Scientific Knowledge: Phytoconstituents and Their Roles in Lung and Colorectal Cancer Signaling Pathways

**DOI:** 10.3390/biom15030380

**Published:** 2025-03-05

**Authors:** Ilma Imtiaz, Janet Schloss, Andrea Bugarcic

**Affiliations:** National Centre for Naturopathic Medicine, Faculty of Health, Southern Cross University, Military Road, Lismore, NSW 2480, Australia; i.imtiaz.11@student.scu.edu.au (I.I.); janet.schloss@scu.edu.au (J.S.)

**Keywords:** cancer, traditional medicine, phytocompounds, phytoconstituents, signaling pathways, herbal medicine

## Abstract

Natural plant products have been used for cancer treatment since ancient times and continue to play a vital role in modern anticancer drug development. However, only a small fraction of identified medicinal plants has been thoroughly investigated, particularly for their effects on cellular pathways in lung and colorectal cancers, two under-researched cancers with poor prognostic outcomes (lung cancers). This review focuses on the lung and colorectal cancer signaling pathways modulated by bioactive compounds from eleven traditional medicinal plants: *Curcuma longa*, *Astragalus membranaceus*, *Glycyrrhiza glabra*, *Althaea officinalis*, *Echinacea purpurea*, *Sanguinaria canadensis*, *Codonopsis pilosula*, *Hydrastis canadensis*, *Lobelia inflata*, *Scutellaria baicalensis*, and *Zingiber officinale*. These plants were selected based on their documented use in traditional medicine and modern clinical practice. Selection criteria involved cross-referencing herbs identified in a scoping review of traditional cancer treatments and findings from an international survey on herbal medicine currently used for lung and colorectal cancer management by our research group and the availability of existing literature on their anticancer properties. The review identifies several isolated phytoconstituents from these plants that exhibit anticancer properties by modulating key signaling pathways such as PI3K/Akt/mTOR, RAS/RAF/MAPK, Wnt/β-catenin, and TGF-β in vitro. Notable constituents include sanguinarine, berberine, hydrastine, lobeline, curcumin, gingerol, shogaol, caffeic acid, echinacoside, cichoric acid, glycyrrhizin, 18-β-glycyrrhetinic acid, astragaloside IV, lobetyolin, licochalcone A, baicalein, baicalin, wogonin, and glycyrol. Curcumin and baicalin show preclinical effectiveness but face bioavailability challenges, which may be overcome by combining them with piperine or using oral extracts to enhance gut microbiome conversion, integrating traditional knowledge with modern strategies for improved outcomes. Furthermore, herbal extracts from *Echinacea*, *Glycyrrhiza*, and *Codonopsis,* identified in traditional knowledge, are currently in clinical trials. Notably, curcumin and baicalin also modulate miRNA pathways, highlighting a promising intersection of modern science and traditional medicine. Thus, the development of anticancer therapeutics continues to benefit from the synergy of traditional knowledge, scientific innovation, and technological advancements.

## 1. Introduction

Cancer is a significant global health issue and is one of the leading causes of death worldwide [1]. It is a heterogeneous group of diseases characterized by alterations in multiple cellular signaling pathways, including the activation of oncogenes and the inactivation of tumor suppressor genes. These alterations lead to abnormal cell cycle progression and the evasion of apoptosis, which are key hallmarks of cancer [2]. In addition to evading apoptosis, cancer cells acquire other hallmarks that contribute to their growth and progression, such as self-sufficiency in growth signaling, sustained angiogenesis, evasion of immune detection, tissue invasion, and eventually metastasis [3,4]. While there are many different types of cancers, lung cancer is one of the most common and has a high global mortality rate, closely followed by colorectal cancer [5,6].

The development of cancer involves a complex series of events affecting various signaling pathways related to cell growth, differentiation, and programmed cell death. Dysregulation of these pathways contributes to both the initiation and progression of cancer. For example, in non-small cell lung cancer (NSCLC), dysregulation of the Ras–Raf–MEK–ERK, PI3K/Akt, ERK, STAT3, and EGFR pathways has been implicated [for a recent review, see [7]. Among these, the PI3K/Akt/mTOR pathway is dysregulated in 50–70% of NSCLC cases and approximately 47% of squamous cell carcinomas (SCCs), as reported by the Cancer Genome Atlas project [8]. Additionally, activating mutations in EGFR, KRAS, PI3K, or Akt, PIK3CA amplification, or loss of negative regulation by tumor suppressor gene PTEN can lead to changes in lung cells [8].

Similarly, alterations in the EGFR/MAPK, Wnt/β-catenin, PI3K, TGF-β, Notch, and NF-κB pathways have been implicated in colorectal cancer [see review [9]]. Notably, dysregulation of the Wnt/β-catenin pathway in colorectal cancer is often due to inactivating mutations of the APC tumor suppressor or oncogenic mutations of β-catenin [10]. Aberrant Wnt pathway signaling is categorized as an early event in the progression of approximately 90% of colorectal cancers [11]. Downregulation of ferroptosis, a recently identified form of regulated cell death characterized by iron dependence, elevated intracellular Fe^2+^ levels, lipid peroxidation, and glutathione (GSH) depletion, is also identified as a mechanism for CRC development [12]).

Lung and colorectal cancers have been found to be driven by distinct genetic alterations. In colorectal cancer, mutations in the APC gene, a tumor suppressor and key regulator of the Wnt pathway, microsatellite instability (MSI) from mutations in mismatch repair genes MLH1 and MSH2, and activation of the NF-κB pathway and COX-2 expression associated with chronic inflammation are more prevalent [13,14,15]. In contrast, in lung cancer, mutations have been found in oncogenes such as TP53, EGFR, and KRAS, associated with carcinogen exposure during smoking, and hypoxia-induced activation of HIF-1α, promoting angiogenesis, is more frequent [16,17]. The heterogeneity among cancer types arises from various factors, including histopathological classification, molecular and genetic characteristics, driver gene expression, intra- and inter-tumor variability, and the tumor microenvironment [18,19,20,21]. Understanding and characterizing this heterogeneity is crucial for developing targeted and personalized approaches for the diagnosis and treatment of different types of cancer.

Conventional treatments for lung and colorectal cancer include surgery, radiation therapy, chemotherapy, targeted therapy, and immunotherapy [22]. Despite the advancements in conventional treatments, challenges occur and can include adverse reactions, therapy resistance, limited efficacy in advanced stages, and impact on the patients’ quality of life [23]. Herbal medicines have shown promise in enhancing conventional cancer treatments and improving patient outcomes by sensitizing cancer cells to pharmaceutical cancer agents, improving patient survival, reducing chemotherapy-induced side effects, and enhancing the quality of life for patients with lung and colorectal cancer [24].

Phytochemicals, the biologically active compounds naturally occurring in plants, have been shown to exhibit anticancer properties by inducing selective toxicity in proliferating cells, reducing oxidative stress, modulating the cell cycle, inhibiting angiogenesis, and inducing cell death [4]. For instance, the leaf extract of *Annona muricata* (soursop), widely used in traditional medicine for treating various ailments and diseases, particularly cancer and parasitic infections, has shown anticancer activity in lymphoma cells [25]. The saponins found in the flowers of *Camellia sinensis* (tea plant), traditionally used by Chinese and Indian practitioners as a stimulant, diuretic, astringent, and for heart health, inhibit cell growth and induce apoptosis in ovarian cancer cell lines [26,27]. The bark of *Marsdenia cundurango* (cundurango) has been traditionally used in the treatment of gastrointestinal (GI) cancers affecting the mouth, esophagus, and stomach. It is generally administered as a decoction, tincture, or fluid extract to relieve pain and enhance digestive and nervous system functions [28]. Additionally, the isolated pregnane glycosides from this plant have demonstrated tumor-selective cytotoxicity in human leukemia cells [29], while silibinin derived from *Silybum marianum* (milk thistle) demonstrated hepatoprotective and antitumor effects both in vitro and in vivo by reducing oxidative stress and inhibiting proliferation in hepatocellular carcinoma [30].

While several reviews have addressed the phytocompounds and phytoconstituents found in traditional medicinal plants and their anticancer activities [31,32,33], no review has focused specifically on interactions between phytoconstituents and molecular pathways in lung and colorectal cancers. Thus, this review examines the key bioactive phytoconstituents of eleven traditional medicinal plants—*Curcuma longa, Astragalus membranaceus, Glycyrrhiza glabra, Althaea officinalis, Echinacea purpurea, Sanguinaria canadensis, Codonopsis pilosula, Hydrastis canadensis, Lobelia inflata, Scutellaria baicalensis,* and *Zingiber officinale*—and their modulation of lung and colorectal cancer signaling pathways. These plants were selected based on a scoping review that collated written information on traditional medicine used in cancer management from 1800 to 2021 [28] and an international survey of herbs used in current clinical practice for these cancers [34] from our research group. The herbs identified in both the survey and the scoping review with existing literature on anticancer properties were chosen for this review. The review highlights the active phytoconstituents present in these herbs, their regulation of cellular signaling pathways, traditional uses and preparations, and ongoing pre-clinical and clinical research.

## 2. Key Signaling Pathways Involved in Lung and Colorectal Cancer

**Figure 1:**
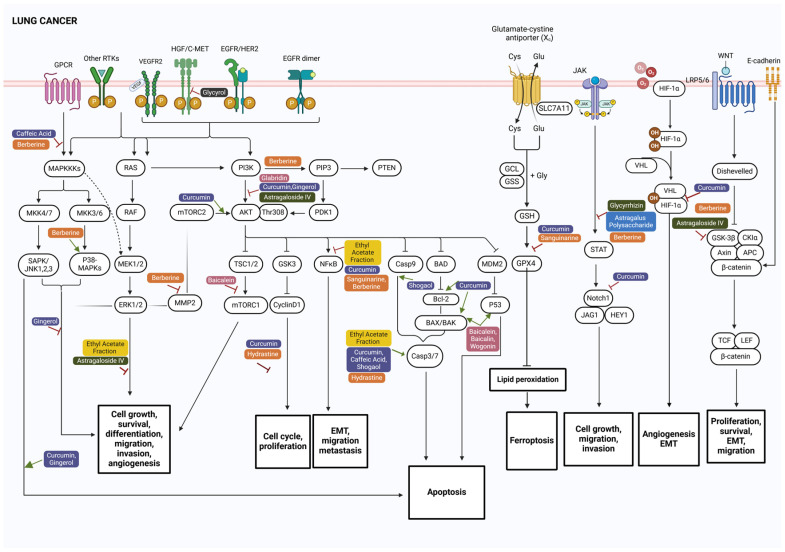
Cellular signaling pathways induced/inhibited by phytoconstituents in lung cancer. Binding of ligands to RTKs triggers the activation of various downstream signaling pathways in lung cancer. These pathways, including the PI3K/Akt/mTOR, RAS/RAF/MEK/ERK, JAK/STAT, and Wnt/β-catenin pathways, collectively drive such processes as cell growth, proliferation, and metastasis in lung cancer. The PI3K/Akt/mTOR pathway inhibits proapoptotic proteins, while the RAS/RAF/MEK/ERK pathway activates proto-oncogenes, and the JAK/STAT pathway induces pro-survival oncogenes. The pathways are intertwined and play crucial roles in the progression of lung cancer. The signaling molecules and effector proteins within these pathways are potential targets for phytochemicals aiming to intervene in lung cancer progression. Created with BioRender.com. Legend: -|—inhibit, →—induce. The different colors represent alkaloids (orange), phenolic compounds (purple), terpenoids and steroids (green), flavonoids (pink), polysaccharides (blue), whole fractions (yellow), and coumarins (black).

Lung cancer is a product of aberrations in normal cell function, including oxidative stress, genetics, and multiple signaling pathways. Among these, the MAPK and PI3K/Akt pathways are well-characterized in lung cancer. The MAPK cascade, initiated by receptor tyrosine kinases (RTKs) such as EGFR, activates RAS proteins, leading to the activation of RAF, MEK1/2, and ERK1/2, which regulate genes involved in proliferation, differentiation, and apoptosis. Mutations in oncogenes such as EGFR, HER2, KRAS, or BRAF can lead to sustained activation of the MAPK/ERK pathway [35]. These transmembrane receptors also trigger various signaling cascades that ultimately activate pro-survival oncogenes such as XIAP, Mcl-1, survivin, and Bcl-2 while inactivating proapoptotic genes such as FOXO, ultimately promoting cell proliferation, survival, and cell cycle progression [7]. Key signaling pathways involved in the pathogenesis of lung cancer include RAF/MEK/ERK, PI3K/Akt/mTOR, and JAK/STAT signaling (Figure 1) [36,37]. Additionally, in NSCLC, the p53 pathway is often downregulated, while the Wnt, EGFR, and NF-κB pathways are upregulated. The PI3K/Akt pathway promotes proliferation, while the ERK and STAT pathways, regulated by the EGFR pathway, are associated with the inhibition of apoptosis and chemotherapy resistance [7].

**Figure 2:**
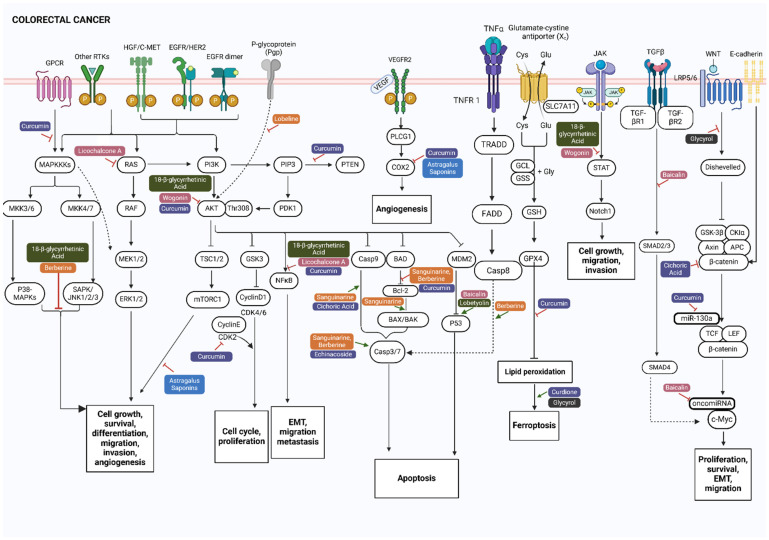
Cellular signaling pathways induced/inhibited by phytoconstituents in colorectal cancer. The activation of downstream signaling pathways in CRC occurs upon ligand binding to RTKs. These pathways, such as MAPK, HER2, PI3K/AKT/mTOR, HGF/c-Met, p53, Wnt/β-catenin, JAK/STAT, TGFB, TNF-α, and NF-κB, collectively regulate cellular processes driving cancer cell cycle progression, proliferation, migration, and invasion while inhibiting metastasis. Effector proteins and signaling molecules within these pathways represent potential targets for phytochemical interventions aimed at impeding colorectal cancer progression. Created with BioRender.com. Legend: -|—inhibit, →—induce. The different colors represent alkaloids (orange), phenolic compounds (purple), terpenoids and steroids (green), flavonoids (pink), coumarins (black), and polysaccharides (blue).

Colorectal cancer (CRC) develops from abnormal cell growth in the colon or rectum, eventually leading to a cancerous mass. Key risk factors include overweight, obesity, sedentary lifestyle, smoking, alcohol consumption, and dietary habits. These environmental factors may induce genetic changes and epigenetic modifications that may silence tumor suppressor genes or activate oncogenes that contribute to CRC development [38]. Such changes can activate several signaling pathways that promote tumor progression, including the Wnt, PI3K/Akt/mTOR, MAPK, TGF-β, and p53 pathways (Figure 2) [38,39]. Additionally, non-coding RNAs such as microRNAs (miRNAs), long non-coding RNAs (lncRNAs), and circular RNAs (circRNAs) play a crucial role in regulating these pathways, with their expression changes linked to CRC progression [39]. Other dysregulated pathways include receptor tyrosine kinase (RTK) signaling, which involves such members as vascular endothelial growth factor (VEGF), epidermal growth factor receptor (EGFR), insulin-like growth factor 1 receptor (IGF1R), and MET, as well as apoptotic signaling [40,41]. The mutations that activate various RTKs and affect downstream components of RTK-activated signaling pathways lead to increased cell proliferation, survival, invasion, and metastasis [40,41,42].

In colorectal cancer (CRC), genetic alterations frequently disrupt the Wnt/β-catenin signaling pathway, which is altered in over 90% of CRC cases, emphasizing its significance as a therapeutic target [43]. This pathway is crucial for maintaining cell homeostasis and embryonic development and is linked to tumor cell proliferation, apoptosis, invasion, stemness, and chemotherapy resistance. It also contributes to cancer stem cell (CSC) expansion, angiogenesis, epithelial–mesenchymal transition (EMT), and tumor immunomodulation and is involved in tumor recurrence after treatment, metastasis, and poor patient outcomes [44]. In CRC, EMT is associated with a more invasive and metastatic phenotype and significantly impacts chemotherapeutic resistance. Enhanced Wnt/β-catenin signaling elevates Snail family transcriptional repressor 1 (SNAIL), which suppresses E-cadherin and regulates EMT, thus promoting local invasion [44]. Loss of membranous β-catenin has also been linked to poor prognosis in CRC [43,45,46,47]. Additionally, mutations in EGFR downstream signaling pathways, including KRAS, BRAF, and PIK3CA, are crucial for metastatic CRC progression [48], while abnormalities in the JAK2/STAT3 pathway disrupt apoptosis. The high expression of JAK/STAT3 proteins in both tumor and stromal cells is associated with poor outcomes in CRC [49]. Moreover, inducing ferroptosis in CRC may target cancer cells resistant to other forms of cell death [50]. Key characteristics of ferroptosis include abnormal iron metabolism, lipid peroxidation, System Xc^−^ inhibition, disrupted glutathione/GPX4 balance, and activation of the p53 pathway [51]. Ferroptosis is mediated through signaling pathways such as Nrf2, AMPK, STAT3, p53, and SAPK/JNK [see review [50]] and may also overcome drug resistance, improving CRC prognosis [51].

## 3. Anticancer Phytocompounds and Their Active Constituents

Phytochemicals have gained considerable attention for their potential in targeting cancer pathways, especially in lung and colorectal cancer. They are of interest for their selective toxicity towards cancerous or precancerous cells, effectiveness against various cancer types, oral administration feasibility, and acceptance by the human population [52]. Alkaloids, coumarins, flavonoids, glycosides, terpenoids, steroids, xanthones, lignans, phenylpropanoids, isoprenoids, and sugars are a diverse range of pure compounds sourced from plants and consist of various phytoconstituents. Phytoconstituents such as curcumin, resveratrol, quercetin, and genistein have been extensively studied for their chemopreventive and anticancer mechanisms, demonstrating antioxidant, anti-inflammatory, and anticarcinogenic properties that inhibit cancer progression [53,54,55,56]. Furthermore, plant-derived phytoconstituents such as berberine, curcumin, and sanguinarine have shown potential in regulating ferroptosis (an iron-dependent programmed cell death) through pathways such as GPX4, FSP1, and iron metabolism [57,58,59,60]. This diversity highlights the therapeutic potential of phytochemicals in targeting multiple signaling pathways implicated in the development and progression of lung and colorectal cancer.

**Figure 3:**
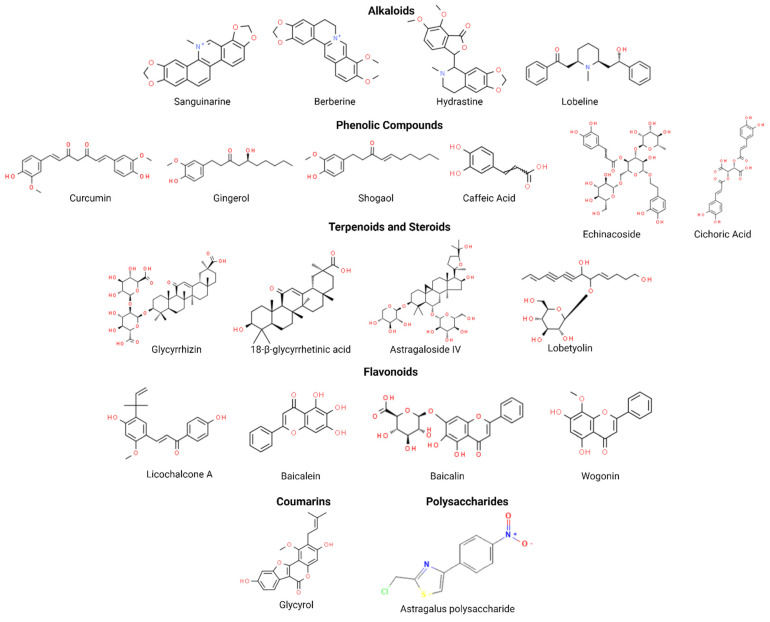
Chemical structures of the anticancer phytoconstituents covered in the current review. Chemical structures included in this paper are from ChemSpider. Created with BioRender.com.

Traditional practices underpinning the generation of empirical knowledge offer valuable cultural and historical insights that enrich the understanding of these phytocompounds. Unlike isolated constituents, traditional medicine preparations often involve whole plants, parts, or mixtures, prepared as a decoction, tincture, fluid extract, or powder, which provide a context for synergistic effects that enhance therapeutic outcomes. While some plant constituents are well-studied, traditional preparations may include less-known compounds that contribute to their overall effectiveness, potentially acting through distinct mechanistic pathways. Integrating tradition with science allows deeper exploration of how these constituents interact within the body, guiding modern drug development. In this narrative review, we review 11 traditional medicinal plants containing 20 key phytoconstituents, with existing literature that supports their ability to regulate cellular signaling pathways in lung and/or colorectal cancer (Table 1). These phytoconstituents belong to the following phytocompound groups: alkaloids (4 constituents), phenolic compounds (6 constituents), terpenoids and steroids (4 constituents), and flavonoids (4 constituents), with the remaining being coumarins and polysaccharides (1 constituent each). The chemical structures of these constituents are presented in Figure 3.

### 3.1. Alkaloids

Alkaloids are a diverse class of nitrogen-containing, low molecular weight compounds found in various plant parts, including seeds, roots, and leaves. They encompass multiple structural types, such as indole, pyrrole, and isoquinoline alkaloids [61]. Traditionally, alkaloids were extracted using methods such as maceration (soaking in solvents), decoction (boiling water), and infusion (hot water) [62,63]. Modern techniques, such as supercritical fluid extraction (SFE), ultrasound-assisted extraction (UAE), and microwave-assisted extraction (MAE), are now used to enhance extraction efficiency and accuracy [64]. Approximately half of anticancer drugs, including vinblastine, vincristine, camptothecin, and paclitaxel, are plant-derived alkaloids and have traditional uses among indigenous peoples [65,66]. This prevalence in plant-based drug discovery could be due to their long history of ethnomedical use, unique chemical properties, and biological activities, which may target multiple cancer-related signaling pathways and have fewer adverse effects compared to synthetic drugs [67,68,69,70]. Plant-derived alkaloids sanguinarine, berberine, hydrastine, and lobeline have shown anticancer properties, and the plants containing these alkaloids have traditionally been used in cancer treatment as whole herbal preparations [28].

Sanguinarine, a benzophenanthridine alkaloid, targets multiple pathways in lung cancer (Table 1, Figure 1). It induces ferroptosis in NSCLC cells by modulating STUB1-mediated GPX4 ubiquitination, leading to increased oxidative stress and reduced cell proliferation and metastasis [60]. Additionally, it inhibits macrophages through the NF-κB pathway, affecting exosome regulation and thereby decreasing cancer metastasis and proliferation [71]. Similarly, berberine exerts antimetastatic effects by reducing migration and invasion of A549 cells by inhibiting the NF-κB pathway and promotes ferroptosis when combined with other inducers through the p53-dependent SLC7A11–GPX4 pathway [57,72]. Hydrastine, specifically, (-)-β-hydrastine, reduces proliferation and induces apoptosis in NSCLC and SCLC cells by downregulating expression of cell cycle regulators cyclin D1/D3 and CDK2/4/6 and activating the mitochondrial apoptosis pathway [73].

Interestingly, sanguinarine and berberine induce apoptosis in colorectal cancer cells by targeting different pathways, as illustrated in Figure 2. Sanguinarine acts through the intrinsic pathway by activating caspase 3 and caspase 9, effectively inhibiting tumor growth without toxicity [74]. Berberine, via the extrinsic pathway, generates ROS and activates the JNK/p38 MAPK pathway and FasL, leading to caspase 3 and caspase 8 activation, cytochrome C release, and the downregulation of antiapoptotic proteins [75].

While sanguinarine and berberine target apoptosis pathways, other alkaloids such as lobeline show potential through distinct mechanisms, such as overcoming multidrug resistance. It demonstrated potential to inhibit P-glycoprotein (P-gp) and enhance the efficacy of chemotherapeutic agents such as mitoxantrone, doxorubicin, and rhodamine 123 in Caco2 cells [76]. Lobeline-containing plants, including *Lobelia* species such as *Lobelia inflata* (Indian tobacco) and *Lobelia tupa* (devil’s tobacco), have traditionally been used for respiratory conditions and, occasionally, for digestive cancers [77,78]. Despite this, no studies have reported their anticancer activity in lung cells. At toxic doses, lobeline exhibits paralytic effects, necessitating caution in its use [79]. Although Lobelia’s inclusion in smoking cessation products and complementary medicine products was banned in 1993 due to ineffectiveness and strong toxicity, it remains available as a homeopathic remedy through qualified practitioners in Australia [77,80,81,82].

Beyond their mechanistic actions, many alkaloids, including berberine and sanguinarine, have a rich ethnobotanical history, showcasing their traditional use in cancer treatment. For instance, berberine, found in several plants including *Berberis* species and *Mahonia aquifolium* (Oregon grape) and, most notably, *Hydrastis canadensis* (goldenseal), has traditionally been extracted in tinctures and fluid extracts of *Hydrastis canadensis* and used for cancer treatment, as well as for such ailments as ulcers and digestive issues [83,84]. Similarly, sanguinarine, along with such alkaloids as sanguirubine and chelerythrine, is derived from *Sanguinaria canadensis* (blood root). Traditionally, the powdered root and tinctures of this plant have been applied to tumors and rectal cancers, targeting the mucous membranes that protect the rectal lining [28,85,86]. However, despite these traditional applications, *Sanguinaria canadensis* has faced controversy due to its toxicity and clinical limitations [87,88]. This highlights the challenges in translating ethnobotanical knowledge into safe and effective therapies, where factors such as plant growth conditions, geographical location, and variations in preparation and processing methods could influence safety and efficacy.

### 3.2. Phenolic Compounds

Phenols are organic compounds with one or more hydroxyl (-OH) groups attached to an aromatic ring (Figure 3) and are found in a variety of foods such as vegetables, fruits, spices, grains, legumes, and nuts [89]. Traditionally, phenols were extracted using such methods as maceration, decoction, percolation, infusion, digestion, and Soxhlet extraction, but these techniques are less common due to their large solvent use, long extraction times, and high temperatures that can degrade phenolic compounds. In contrast, modern methods, such as supercritical fluid extraction (SFE), microwave-assisted extraction (MAE), ultrasound-assisted extraction (UAE), pressurized liquid extraction (PLE), and subcritical water extraction (SWE), are more efficient, using moderate temperatures, shorter extraction times, and safer solvents to produce phenolic-rich extracts [90]. Among the many phenolic compounds, curcumin, gingerol, and caffeic acid derivatives have a long history of use in traditional medicine. Both whole plant preparations and isolated compounds of phenols have been studied for their anticancer properties.

Curcumin, a major phenolic compound in *Curcuma longa* (turmeric), demonstrates versatility by targeting multiple pathways in different cancer models. In lung cancer, curcumin inhibits angiogenesis and proliferation while promoting apoptosis by modulating the MAPK, PI3K/Akt, and NF-κB pathways [91]. It blocks VEGF signaling to suppress angiogenesis, enhances apoptosis via caspase 3, and targets apoptosis pathways by activating p38 [92,93]. It also inhibits the proliferation of SW480 colon cancer cells through the Wnt/β-catenin pathway and downregulates miR-130a [94]. Interestingly, in CRC models, curcumin also induces apoptosis, but via distinct mechanisms: by inducing ferroptotic cell death through autophagy by increasing ACSL4 protein levels and reducing SLC7A11 and GPX4 protein levels. This process is characterized by mitochondrial membrane rupture, reduced mitochondrial cristae, and increased autolysosomes [95]. These contrasting mechanisms highlight curcumin’s broad utility across different cancer types (Figure 1 and Figure 2). In contemporary naturopathic practice, curcumin, prescribed in combination with piperine, a black pepper compound that enhances curcumin’s bioavailability [96], is used to support lung cancer patients by reducing circulating tumor cells and providing nutritional benefits [97,98].

Gingerols, derived from *Zingiber officinale* (ginger), have traditionally been used to treat gastrointestinal issues, metabolic diseases, and arthritis, and are included in Chinese and Korean formulations for lung cancer [99,100,101,102]. These ginger-derived phenolics induce apoptosis through various pathways in lung cancer. For example, 10-gingerol induces apoptosis and inhibits metastasis by targeting Akt and p38 MAPK [103], while 6-gingerol promotes cell death via ferroptosis and autophagy, and 6-shogaol induces cell death through the p53 pathway [104]. Notably, ginger-derived shogaols exhibit greater potency than gingerols in inhibiting cancer cell growth. For example, 6-shogaol exerts a stronger effect on arachidonic acid release and nitric oxide synthesis in both CRC and lung cancer cells [105]. In addition to individual constituents, whole-plant part extracts of *Zingiber officinale* have also been studied. For instance, ginger leaf extract promotes apoptosis in HCT116, LoVo, and SW480 colorectal cancer cells by upregulating ATF3 and modulating ERK1/2 [106]. Bismuth oxide nanoparticles synthesized from ginger extract selectively target HCT116 cells through the PI3K/Akt/mTOR pathway [107]. While leaf extracts show promising results, root extracts—traditionally used for ethnomedicinal purposes—could also be of interest for their potential anticancer properties in vitro [108,109].

Caffeic acid, a key phenolic compound, regulates CRC cell proliferation, migration, and apoptosis through the MAPK pathway [110,111]. Together with echinacoside and cichoric acid, caffeic acid is present in *Echinacea purpurea*, a plant native to North America. Echinacoside induces apoptosis and cell cycle arrest in SW480 colon cancer cells through oxidative DNA damage and increased active caspase 3 [112], while cichoric acid triggers apoptosis in Caco2 and HCT116 cells, indicated by DNA fragmentation and caspase 9 activation [110,111].*Echinacea purpurea* also contains bioactive compounds, such as caffeic acid derivatives and polysaccharides, primarily in its flowers. Traditionally used in cancer management, *Echinacea purpurea* is known for its immune-boosting properties and potential cytotoxic effects against colon cancer [28,113,114,115].

Interestingly, complex mixtures of whole plant extracts, obtained through different extraction methods, also exert anticancer effects (Table 1). For instance, the hexanic extract of dried *Echinacea purpurea* roots, rich in alkamides and caffeic acid derivatives, reduces COLO320 colon cancer cell viability through apoptosis induction [116]. Similarly, in HCT116 colorectal cancer cells, a 50% ethanol extract of *Echinacea purpurea* flowers triggers apoptosis by reducing telomerase activity [115]. In contrast, studies in lung cancer models, such as the A549 non-small cell lung cancer (NSCLC) line, reveal that the dichloromethane root extract of *Echinacea purpurea* reduces cell viability, induces early apoptosis, and causes cell cycle arrest [117]. Beyond apoptosis, intradermal administration of *Echinacea purpurea* has been shown to inhibit angiogenesis in lung cancer models, highlighting its potential to target different pathways based on the cancer type.

### 3.3. Terpenoids and Steroids

Terpenoids refer to the class of compounds that contain many isoprene structural units, and based on the number of isoprene units, their classification ranges from monoterpenes to polyterpenes. Apart from the terpene hydrocarbon form, terpenoids are also present as oxygen-containing derivatives such as alcohols, glycosides, aldehydes, ketones, carboxylic acids, and esters [118]. They are recognized for their anti-inflammatory, anticancer, antimicrobial, and hypoglycemic properties [119]. Terpenoids such as glycyrrhizin, 18-β-glycyrrhetinic acid, astragaloside IV, and lobetyolin (Figure 3), derived from traditional medicinal plants, modulate various cellular signaling pathways to exert anticancer effects (Figure 1 and Figure 2).

*Glycyrrhiza glabra* (licorice), a key component of Ayurvedic and Chinese medicine for over 400 years, is traditionally used to treat a variety of conditions, including wound healing, edema, and intrinsic hemorrhage. Its roots contain such bioactive compounds as glycyrrhizin, glycyrrhetic acid, glycyrrhetinic acid, and licochalcones, which exhibit anticancer properties [120,121]. In traditional Korean medicine, *Glycyrrhizae sp.* is included in herbal formulas such as Gunchil-dan and Bangam-tang, contributing to prolonged progression-free survival in cancer patients [122]. Glycyrrhizin, a triterpenoid saponin with anti-inflammatory and immunomodulatory properties, has demonstrated anticancer effects in lung cancer and in tumor-bearing mice. Glycyrrhizin, alone or combined with cisplatin, inhibited thromboxane A2 and reduced the proliferating cell nuclear antigen (PCNA) expression, suggesting its potential in overcoming chemotherapy resistance [123]. Additionally, in tumor-bearing mice, glycyrrhizin suppressed lung cancer growth by inhibiting the HMGB1 and JAK/STAT pathway activity, both of which are involved in cancer cell migration and invasion [124]. Moreover, astragaloside IV (AS-IV), a saponin from Astragali radix, inhibits lung cancer metastasis and in A549 NSCLC cells, it reduces migration and invasion, likely through the PKC-α–ERK1/2–NF-κB pathway [125]. It also targets the Akt/GSK-3β/β-catenin signaling axis, crucial for cancer cell proliferation and survival, further inhibiting NSCLC progression [126].

Apart from the isolated constituents, the ethyl acetate fraction (EAM) from *Astragalus membranaceus*, rich in ploysaccharides, flavonoids, and saponins (a subclass of terpenes), reduces NSCLC cell proliferation in a dose- and time-dependent manner by inducing apoptosis through both intrinsic and extrinsic pathways and by inhibiting the ERK pathway [127]. *Codonopsis pilosula* (dangshen), widely used in Chinese herbal medicine, is traditionally administered to improve immunity and reduce therapy-induced fatigue in cancer patients. Its key bioactive compounds, including lobetyolin and atractylenolide III, are recognized for their anticancer effects, particularly in inducing ferroptosis in lung cancer cells [128,129]. Lobetyolin, a polyacetylene glycoside, primarily from *Codonopsis pilosula* and *Lobelia inflata*, enhances cisplatin efficacy in lung cancer models by inhibiting the epithelial–mesenchymal transition [130]. Lobetyolin also induces apoptosis in HCT116 CRC cells by inhibiting ASCT2-mediated glutamine metabolism, potentially regulated by p53 [131].

In a colorectal cancer model induced by azoxymethane (AOM)/dextran sodium sulfate (DSS), glycyrrhizin inhibits pathogenesis by targeting the HMGB1–TLR4–NF-κB signaling pathway, reducing DNA damage and cancer stem cell proliferation [132]). The glycyrrhizin metabolite, 18-β-glycyrrhetinic acid, shows anticancer effects in colorectal cancer by suppressing PI3K and STAT3 signaling, leading to reduced cell proliferation, migration, and invasion in LoVo, SW480, and SW620 cells [133]. Similarly to the constituents, the crude extracts of herbs have been studied in colorectal cancer models (Table 1). For example, an ethanol extract of *Glycyrrhiza glabra* induces apoptosis in HT29 cells via HSP90 downregulation [134], while total saponins extracted from *Astragalus membranaceus* root modulate mTOR and COX-2 signaling, reducing VEGF levels and suppressing angiogenesis in HCT116 colon cancer cells [135]. The whole root extract of *Astragalus membranaceus* also inhibits colon cancer proliferation, induces cell cycle arrest, promotes apoptosis, and reduces migration by modulating the PI3K/Akt pathway and regulating miR-590, an oncomiR associated with various tumors [136]. Additionally, *Astragalus membranaceus* (whole herbal extract) and its isolated isoflavonoids, formononetin and calycine, decrease cell viability and proliferation and promote apoptosis in Caco2 and HT29 colon cancer cells by inhibiting the ERK1/2 pathway [137]. Collectively, this highlights the therapeutic potential of key flavonoids and their source herbs, *Glycyrrhiza glabra*, *Astragalus membranaceus,* and *Codonopsis pilosula*, providing a basis for further preclinical and clinical studies to assess their efficacy in cancer management.

### 3.4. Flavonoids

Flavonoids, the largest group of phenolic phytocompounds, are abundant in leaves and fruit peels and are key in cancer prevention and treatment due to their diverse bioactivities. Their structure consists of fifteen carbon atoms arranged in two benzene rings linked by a heterocyclic ring containing oxygen. Subgroups of flavonoids include flavones, flavonols, isoflavones, flavanones, flavans, and anthocyanidins [138]. Traditionally, Soxhlet extraction was favored for flavonoid extraction due to its simplicity and cost effectiveness. However, modern techniques such as supercritical fluid extraction (SFE), microwave-assisted extraction (MAE), and ultrasound-assisted extraction (UAE) have improved efficiency and yield, becoming the methods of choice for extracting flavonoids and other phenolic compounds [138].

Among flavonoids with anticancer properties, licochalcone A from *Glycyrrhiza glabra* (licorice) and baicalein, baicalin, and wogonin from *Scutellaria baicalensis* (skullcap) exhibit multifaceted mechanisms of action. Licochalcone A inhibits CRC cell proliferation and induces apoptosis in HCT116 cells via the NF-κB and Ras/Raf/MEK pathways [139]. In lung cancer models, it blocks cell migration and invasion in A549 and H460 cells by downregulating MMP-1 and MMP-3 [140]. In lung cancer models, it blocks cell cycle progression, reducing proliferation by lowering levels of MDM2, cyclin B1, Cdc2, and Cdc25C [141]. Baicalein reduces cell cycle progression and angiogenesis in H460 NSCLC cells by downregulating VEGF and FGFR-2, two key drivers of angiogenesis [142]. Baicalin also demonstrates dual effectiveness: in H1299 lung cancer cells, it induces Akt-dependent apoptosis by suppressing the Akt/mTOR pathway [143], while in HT-29 colon cancer cells, it triggers apoptosis by inhibiting c-Myc and oncomiRNAs [144]. Additionally, baicalin arrests the G1 phase of the cell cycle in CRC cells by promoting p53-independent apoptosis and inhibiting the epithelial–mesenchymal transition via TGF-β/Smad pathway suppression [145]. Wogonin further highlights the versatility of skullcap flavonoids by inducing autophagy and apoptosis in SW480 CRC cells, mediated byG2/M phase arrest and the STAT3 and PI3K/Akt pathways suppression, while in lung cancer models, it suppresses A549 and H460 cell growth and metastasis by downregulating MMP1 and modulating the PI3K/Akt pathway [146,147].

In addition to isolated constituents, whole-plant extracts of *Scutellaria baicalensis* offer therapeutic potential. The flavonoid-rich root contains baicalin, baicalein, wogonin, volatile oils, and trace elements [148]. Crude ethanolic extracts of the root induce apoptosis in lung cancer cells through the p53 and BAX pathways [149], while water extracts from Korean *Scutellaria baicalensis* inhibit metastasis by downregulating MMP-2 [150]. Refer to Table 1 for an overview of the constituents present in these whole-plant extracts. Traditional preparations, such as the Qing-re-huo-xue formula combining *Scutellaria baicalensis* and Radix paeoniae rubra, show synergistic effects by activating the p53 and GSK-3β/Nrf2 pathways, inducing ferroptosis and apoptosis in NSCLC [151]. Historically, *Scutellaria baicalensis* has been used in traditional medicine to treat cancer and respiratory infections, often in combination with *Lobelia inflata* and other herbs, for addressing digestive cancers and alleviating nerve irritation [152,153]. These findings highlight the extensive use of the flavonoid-rich root of *Scutellaria baicalensis* across various traditional medical systems and its differing effects as isolated constituents or in herbal preparations in diverse cancer models.

### 3.5. Coumarins

Coumarins, a class of polyphenolic compounds, are predominantly found in plant seeds, roots, and leaves, with some microbial sources. Among these, glycyrol, derived from *Glycyrrhiza glabra* (licorice), has diverse therapeutic properties, including anticancer, anti-inflammatory, antimicrobial, and hepatoprotective activities [154]. Its diverse mechanisms of action in different lung and CRC models show its potential as a candidate for targeted therapies.

In lung cancer models, glycyrol demonstrates its effectiveness by reducing A549 cell growth and overcoming gefitinib resistance in HCC827GR cells. This is achieved through the inactivation of MET within the COX2/MET/TOPK signaling axis, which increases drug sensitivity [155,156]. In contrast, in CRC cells, glycyrol exerts its anticancer effects through a different mechanism. Specifically, it inhibits cell proliferation via the Wnt/β-catenin signaling pathway, induces ferroptosis, and, in combination with butyrate (a gut microbiota-derived short-chain fatty acid), increases apoptosis in HT29 and HCT116 cells by activating caspase 3, a key marker of apoptosis [155,157]. These findings depict glycyrol’s ability to engage distinct signaling pathways depending on the cancer type.

The role of *Glycyrrhiza glabra* in traditional medicine further supports its clinical significance. Often referred to as an “essential herbal medicine” in traditional Chinese medicine (TCM), licorice is believed to reduce toxicity and enhance the efficacy of herbal preparations, as reflected in the TCM adage, “nine out of ten formulas include licorice” [154]. This dual role, both as a therapeutic agent and an adjunct in polyherbal formulations, demonstrates its multifaceted therapeutic potential.

### 3.6. Polysaccharides

Polysaccharides are complex carbohydrates, composed of long chains of monosaccharide units connected by glycosidic bonds (Figure 2). Polysaccharides show promising anticancer activity in various cancer cell lines and may offer selective tumor cell targeting with minimal toxicity [158]. One such example is Astragalus polysaccharide (APS), derived from *Astragalus membranaceus*, which has been shown to enhance immune response, induce apoptosis, and inhibit cancer cell proliferation and metastasis [159].

In the context of lung cancer, APS suppresses the S1PR1/STAT3 signaling pathway, which reduces the premetastatic niche, a distant secondary microenvironment prepared for future metastatic growth, which is essential for preventing metastasis [160]. In contrast, in CRC models, APS induces autophagy in HCT116 tumor tissues and cells, inhibiting proliferation and migration through the PI3K/Akt/mTOR pathway [161]. Furthermore, APS enhances drug sensitivity in cisplatin-resistant HT-29/DDP cells by downregulating miR-20a (Figure 2). This inhibition of miR-20a increases TGFBR2 expression leading to reduced cell proliferation and invasion, as well as the induction of apoptosis [162]. The downregulation of miR-20a, which targets EGR2, and the inhibition of TGFBR2, a tumor suppressor in the TGF-β pathway, highlights the therapeutic potential of APS in CRC [163,164].

The dried root of *Astragalus membranaceus*, known as Astragali radix or huáng qí, is widely used in both TCM and Western herbal medicine [165,166]. In TCM, Astragalus is often combined with other herbs in complex formulations to treat lung cancer, adhering to the traditional philosophy of integrating principal, assistant, adjuvant, and mediating guide herbs for a more comprehensive therapeutic approach [167].

## 4. Current Progress of Clinical Trials of Phytoconstituents in Cancer Therapy

Several of the discussed medicinal plants traditionally used for cancer treatment, along with their phytoconstituents, have been evaluated in preclinical and clinical models. For instance, berberine combined with gefitinib is currently being evaluated in a clinical trial as a first-line treatment in lung adenocarcinoma patients with EGFR mutations (NCT03486496), while berberine hydrochloride is in a clinical trial for colorectal adenoma prevention in patients with a history of colorectal cancer (NCT03281096). Clinical trials are also assessing curcumin’s efficacy in lung (NCT01859858) and colorectal cancer (NCT02321293), highlighting its growing importance in integrative oncology [168,169]. Although licochalcone A is under investigation for oral squamous cell carcinoma (NCT03292822), no trials currently explore its potential in lung or colorectal cancers.

Among medicinal plants, *Zingiber officinale*, is being evaluated in clinical trials for CRC prevention, with one examining its preventive effects (NCT01344538) and another its impact on the gut microbiome in colorectal adenoma (NCT03268655) [170,171]. *Astragalus membranaceus* is under investigation for its potential in treating cancer-related conditions, including trials targeting immune alterations in NSCLC patients (NCT01802021) and preventing oxaliplatin-induced neuropathy in stage IIa–IIIc CRC patients (NCT04690283) [172].

While *Echinacea purpurea* is not being evaluated for cancer, *Echinacea angustifolia* is studied as a part of a botanical therapy for mucositis treatment in head and neck cancer patients (NCT01674374). Licorice root extract is studied in metastatic prostate cancer (NCT00176631), but not in lung or colorectal cancers, despite preclinical evidence. Additionally, *Codonopsis pilosula* is being evaluated for its efficacy in combination therapies, such as with adjuvant chemotherapy in pancreatic cancer (NCT05613465) and in reducing recurrence of colorectal adenomatous polyps (NCT03616444). Further, ginger extract containing 20% 6-shogaol is being evaluated for its potential to improve cytopenias (blood markers) in patients with lower-risk myelodysplastic syndromes (ACTRN12623001349639). These clinical trials highlight the benefits of these herbs as extracts from specific plant parts of *Echinacea, Codonopsis, Glycyrrhiza,* and *Zingiber* species, identified as effective against colorectal and/or lung cancers, but it is important to note that it is not clear if these trials are also using traditional herbal preparations or are extracting the perceived active phytoconstituent. On the other hand, clinical research gaps clearly exist for several herbs. For example, despite their traditional uses and in vitro anticancer properties, phytoconstituents from *Sanguinaria canadensis, Lobelia inflata, Althaea officinalis,* and *Scutellaria baicalensis* have not been clinically evaluated for any cancer type (https://clinicaltrials.gov/ (accessed on 10 June 2024)).

**Table 1:** Molecular mechanisms and anticancer activities of traditional medicinal plant extracts and phytoconstituents in lung and colorectal cancer.

Plant Species	Crude Extracts Tested	Isolated Compounds Tested	Anticancer Activity	Target (Protein/Pathway/miRNA)	Cancer Type	Reference
*Curcuma longa*	Not tested	Curcumin	Regulates angiogenesis, induces apoptosis, inhibits proliferation, suppresses cell division, activates autophagy	MAPK, p53, JAK/STAT pathways Wnt/β-catenin pathway (Notch, HIF-1 mRNA, VEGF and NF-κB) P13K/AKT signaling pathway (caspase 3 activity, miR-192-5p) P38 MAPK phosphorylation and ROS-DNA damage	Lung	[92,93,95,173,174,175]
	Not tested	Curcumin Curdione	Regulates angiogenesis, inhibits proliferation, induces apoptosis, induces ferroptosis by activating autophagy (curcumin) Induces ferroptosis (curdione)	PPARy, Prp4B, NF-kB, E2F-1, CDK2, Bcl-2, HKII, COX-2, MAPK cell signaling pathway, Wnt/β-catenin pathway (miR-130a expression), PI3-K/PTEN/AKT pathway (EGFR), ↓ GPX4, FSP1 (curcumin) N6-methyladenosine pathway (curdione)	Colorectal	[58,94,98,176,177]
*Astragalus membranaceus*	Ethyl acetate fraction of the root	Astragalus polysaccharide (APS)Astragaloside IV	Prevents the premetastatic niche (APS) Reduces proliferation, induces apoptosis (EAM) Reduces proliferation, survival, and metastasis and invasion (astragaloside IV)	S1PR1/STAT3 pathway (APS) Caspase 8 and 9, ERK pathway (EAM) PKC-α–ERK1/2–NF-κB pathway and AKT/GSK-3β/β-catenin signaling axis (astragaloside IV)	Lung	[125,126,127,160]
	Powder of the whole root decoction Water extract Whole-plant extract	Total saponins isolated through the methanol extract	Reduces proliferation, induces cell cycle arrest, induces apoptosis, reduce migration (powder) Suppresses viability and proliferation, promotes apoptosis (water extract) Suppress angiogenesis (total saponins) Attenuates inflammation, oxidative stress and induces ferroptosis (whole-plant extract)	PI3K/AKT pathway, several mRNAs (specifically miR-590 expression) (powder) ERK1/2 signaling pathway (p-ERK1/2 and p-Akt expression) (water extract) mTOR and COX-2 signaling (VEGF) (total saponins) NF-κB activation and Nrf2 (whole plant extract)	Colorectal	[135,136,137,178]
*Glycyrrhiza glabra*	Not tested	Glycyrrhizin Glabridin	Reduces tumor progression and the resistance and toxicity of cisplatin, reduces migration and invasion (glycyrrhizin) Reduces metastasis, invasion, and angiogenesis (glabridin)	TxA2 pathway and PCNA, JAK/STAT signaling pathway (HMGB1) (glycyrrhizin) FAK/Rho signaling pathway (glabridin)	Lung	[123,124,179]
	Ethanol root extract	18-β-glycyrrhetinic acid Licochalcone A Glycyrol	Reduces proliferation, invasion, and metastasis, induces apoptosis (18-β-glycyrrhetinic acid) Reduces proliferation, chemoprevention, induces apoptosis (root extract) Inhibits proliferation, induces apoptosis (licochalcone A) Inhibits proliferation and modulates ferroptosis (glycyrol)	PI3K and STAT3 signaling pathways (p-PI3K, p-AKT, p-STAT3, p-JNK, p-p38, and p-NF-κB p65) (18-β-glycyrrhetinic acid) HSP90 expression (root extract) NF-κB and Ras/Raf/MEK pathways (p65 and RAS) and programmed cell death ligand-1 (PD-L1) (licochalcone A) Wnt/β-catenin (glycyrol)	Colorectal	[120,135,139,157]
*Althaea officinalis*	Aqueous root extract Aqueous flower extract	Not tested	Reduces cisplatin-induced cytotoxicity and cell proliferation (root extract) Reduces proliferation, anti-inflammatory activity, cytoprotective activity in red blood cells and antioxidant property (flower extract)	Reduce mRNA expression of iNOS (NOS2), IL-1β, TNF-α, IL-6 (flower extract)	Lung	[180,181]
	Aqueous flower extract	Not tested	Reduces proliferation, anti-inflammatory activity, cytoprotective activity in red blood cells and antioxidant property	Reduces mRNA expression of iNOS (NOS2), IL-1β, TNF-α, IL-6	Colorectal	[180]
*Echinacea purpurea*	Dichloromethane root extract Intradermal injection Immunal forte tablets (dried extract of the plant’s juice)	Caffeic acid	Reduces viability, induces early apoptosis (root extract) Inhibits angiogenesis (injection) Stimulates metabolic activity of granulocytes (tablets) Regulates cell proliferation, migration, and apoptosis (caffeic acid)	ROS-induced caspase-dependent apoptosis (root extract) Increase CD16+ and CD56+ NK cells (tablets) MAPK pathway, inhibition of TMEM16A, calcium-activated chloride channel (caffeic acid)	Lung	[110,117,182,183]
	Hexanic root extract 50% ethanol extract of flowers	Cichoric acid Echinacoside	Reduces viability (root extract) Reduce telomerase activity and induce apoptosis (flower extract and cichoric acid) Induces apoptosis, cell cycle arrest, and oxidative DNA damage (echinacoside)	DNA fragmentation, caspase 9 activation, PARP cleavage, and β-catenin downregulation (flower extract and cichoric acid) Increased active caspase 3, cleaved PARP, and G1/S-CDK blocker CDKN1B (p21) upregulation (echinacoside)	Colorectal	[112,115,116]
*Sanguinaria canadensis*	Not tested	Sanguinarine	Facilitates ferroptosis and apoptosis, reduces proliferation, invasion, migration, metastasis	STUB1/GPX4-dependent ferroptosis (↑ Fe^2+^, ROS levels, and MDA, and ↓ GSH and GPX4) NF-κB pathway (↓ p-p65, TNF-α, IL-6, and CCL-2 expression)	Lung	[60,71]
	Not tested	Sanguinarine	Induces apoptosis, inhibits proliferation and metabolism	↑BAX, ↓Bcl-2 Activates caspase 3 and caspase 9	Colorectal	[74,184]
*Codonopsis* *lanceolata*	Water extract of *C. lanceolata* polyacetylenes (CLP)	Not tested	Reduces proliferation and induces apoptosis	Ras/PI3K/AKT pathway (↓ Ras, PI3K, p-AKT, Bcl-2, cyclin D1, and CDK4 expression, and ↑ Bax, GSK-3β, clv-caspase 3, and clv-caspase 9 expression)	Lung	[185]
*Codonopsis* *pilosula*	Not tested	Lobetyolin	Induces apoptosis, enhances the efficacy of chemotherapy (cisplatin)	ASCT2-mediated glutamine metabolism (p53)	Colorectal	[130,186]
*Hydrastis canadensis*	Not tested	Berberine, (-)-β-hydrastine	Inhibits metastasis and invasion (berberine) Reduces proliferation, migration, and invasion and induces apoptosis [(-)-β-hydrastine]	c-jun, c-fos, and NF-κB, ↓ MMP2, u-PA expression, TIMP-2 and PAI regulation (berberine) Mitochondrial apoptosis pathway (↓ cyclin D1/D3 and CDK2/4/6 expression) [(-)-β-hydrastine]	Lung	[72,73]
	Liquid extract of root, leaf	Berberine	Induces apoptosis (berberine) Reduces viability (liquid extract)	↑ROS, JNK/p38 MAPK pathway, and FasL, ↑ caspase 3 and caspase 8, PARP cleavage, and cytochrome C release, ↓ c-IAP1, Bcl-2, and Bcl-XL (berberine) ↓ P-gp function (liquid extract)	Colorectal	[75,187]
*Scutellaria baicalensis*	Ethanolic root extract Water extract Qing-re-huo-xue decoction (QRHX)	Baicalein Baicalin Wogonin	Induce cell cycle arrest and apoptosis (root extract, baicalein, baicalin, and wogonin) Induces autophagy and cell cycle arrest (baicalein) Reduces metastasis and proliferation (water extract) Induces ferroptosis and apoptosis (QRHX)	↑ p53 and BAX (root extract, baicalein, baicalin, and wogonin) MAP4K3/mTORC1/TFEB-dependent autophagy (baicalein) ↓ G1/S transition, cyclin D1, and MMP-2 (water extract) p53 and GSK-3β/Nrf2 (QRHX)	Lung	[149,150,151,188]
	Not tested	Baicalin	Induces apoptosis (baicalin) Induces cell cycle arrest and apoptosis (baicalin) Induces cell cycle arrest, autophagy, and apoptosis (wogonin)	↓ oncomiRNAs (miR-10a, miR-23a, miR-30c, miR-31, miR-151a, and miR-205) and c-Myc expression (baicalin) ↑ p53-independent apoptosis, ↓ TGF-β/Smad pathway (baicalin) ↓ STAT3 and PI3K/AKT (wogonin)	Colorectal	[144,145,147]
*Zingiber officinale*	Phytocompounds extracted from ginger extract	10-gingerol Gingerol 6-shogaol 6-gingerol	Induces apoptosis and inhibits metastasis (10-gingerol) Inhibits proliferation and invasion (gingerol) Induces cell death and reduces proliferation (6-shogaol) Inhibits growth (ginger extract) Induces ferroptosis (6-gingerol)	↓ AKT and p38 MAPK (10-gingerol) ↓ AKT, p38 MAPK, and EGFR (gingerol) ↑ cytochrome C and caspase 3 and caspase 9 (6-shogaol) ↑ USP14 expression, modulates autophagy-dependent pathways (6-gingerol)	Lung	[99,100,104,105,189]
	Leaf extract Phytocompounds extracted from ginger extract Bismuth oxide nanoparticles from Ginger root extract	Not tested	Reduces viability and induces apoptosis (leaf extract) Inhibit growth (extracted phytocompounds) Induce apoptosis (nanoparticles of ginger root extract)	ERK1/2 activation ↑ activating transcription factor 3 (ATF3) (leaf extract) PI3K/AKT/mTOR (nanoparticles of ginger root extract)	Colorectal	[105,106,107]
*Lobelia inflata*	Not tested	Lobeline	Reverse P-glycoprotein (P-gp)-dependent multidrug resistance	P-glycoprotein (P-gp)	Colorectal	[76]

Legend: ↑ is upregulation or overexpression; ↓ is downregulation.

## 5. Discussion

This narrative review summarized the molecular pathways regulated by various phytochemicals—specifically, alkaloids, flavonoids, phenolic compounds, terpenoids, coumarins, and polysaccharides—derived from eleven medicinal plants (*Curcuma longa*, *Astragalus membranaceus*, *Glycyrrhiza glabra*, *Althaea officinalis*, *Echinacea purpurea*, *Sanguinaria canadensis*, *Codonopsis pilosula*, *Hydrastis canadensis*, *Lobelia inflata*, *Scutellaria baicalensis*, and *Zingiber officinale*) identified for their documented traditional use in cancer management and continued application in naturopathic clinical practice. These compounds were found to modulate crucial signaling pathways, including PI3K/AKT/mTOR, RAS/RAF/MAPK, Wnt/β-catenin, and TGF-β (Figure 1 and Figure 2), all of which are vital to cellular processes such as proliferation, differentiation, metastasis, and apoptosis. While several phytoconstituents, such as curcumin and berberine, have advanced to clinical trials [168,169,190], others—sanguinarine, hydrastine, lobeline, gingerol, shogaol, caffeic acid, echinacoside, cichoric acid, glycyrrhizin, 18-β-glycyrrhetinic acid, astragaloside IV, lobetyolin, licochalcone A, baicalein, baicalin, wogonin, glycyrol, and Astragalus polysaccharide—remain limited to preclinical studies. The review further highlights the significance of exploring additive or synergistic interactions among multiple compounds within whole-plant extracts, as evidenced by studies on *Astragalus membranaceus* [127,135,136,137,159,165,166,167].

The PI3K/AKT pathway is a key driver of tumor growth, invasion, and metastasis in cancers, primarily due to its frequent overactivation and associated genetic alterations [191]. As illustrated in Figure 1 and Figure 2, various phytochemical classes, including alkaloids (hydrastine, sanguinarine, and berberine), flavonoids (baicalein, baicalin, wogonin, and glabridin), and phenolic compounds (curcumin, caffeic acid, and shogaol), can effectively target the key nodes of the PI3K/AKT signaling cascade, such as PI3K, AKT, and mTOR, as well as downstream effectors, including GSK-3β, FOXO, and BAD, and suppress cell cycle progression, proliferation, and metastasis while inducing apoptosis in lung cancer. Apart from the PI3K/AKT pathway, they also regulate apoptotic pathways (intrinsic and extrinsic apoptosis and ferroptosis) and Wnt-β-catenin and P-glycoprotein in colorectal cancer (Figure 2). The molecular structures of these compounds may explain their diverse effects by their binding to key kinases in the pathway [192]. For example, flavonoids have polyhydroxylated structures that facilitate interactions with ATP-binding sites; alkaloids, with nitrogen-containing structures and amphiphilic nature, primarily target cell membranes inhibiting enzymatic activity; and phenolic compounds, with phenolic rings and hydroxyl groups, improve redox balance and binding to active sites [192,193,194,195,196]. This suggests that these phytochemicals can influence multiple pathways within a single cancer type and across different cancers and further argues for the use of whole-plant extracts rather than isolated phytochemicals. For instance, flavonoids can counteract drug resistance in cancer cells by disrupting their dependence on glycolysis (the Warburg effect), a metabolic pathway that sustains rapid proliferation and survival [197]. This resistance is associated with upregulation of HIF-1α, a transcription factor that enhances glycolytic metabolism and is further regulated by the PI3K/AKT pathway—one of the key dysregulated signaling pathways in cancer [197,198]. By targeting HIF-1α and PI3K/AKT, certain flavonoids can interfere with the metabolic reprogramming that underlies drug resistance, thereby reducing the survival advantage of cancer cells. Thus, this disruption of glucose metabolism in tumor cells further enhances the anticancer properties of flavonoids. For example, *Scutellaria baicalensis* contains the constituents baicalin, baicalein, and wogonin, each of which has been shown to inhibit the PI3K/AKT signaling pathway by downregulating HIF-1α and glycolysis in CRC cells [199,200,201]. While these molecular insights such as high molecular diversity, low toxicity, and unique biofunctional properties are essential for understanding the anticancer potential of these phytocompounds, the translation of this knowledge into clinical practice is essential.

Despite robust in vitro effectiveness, there are challenges in translation to clinical application due to issues such as poor bioavailability and rapid metabolism, as observed with curcumin, whose systemic concentrations in clinical trials fall short of the therapeutic levels observed in preclinical studies. For example, treatment of A549 lung cancer cells with 50–100 μM of curcumin inhibited proliferation and induced apoptosis, while 30 μM of curcumin induced cell death in 95% of NCI-H460 cells [202,203]. However, clinical trials show that oral administration of curcumin at doses up to 4–8 g/day results in plasma concentrations below 2.5 ng/mL or even lower at 0.41–1.75 μM [203,204]. Such strategies as combining curcumin with piperine (an active compound in black pepper) to inhibit metabolic degradation and glucuronidation and enhance absorption align with traditional practices of administering turmeric with black pepper, highlighting the potential of integrating traditional knowledge with modern research to improve therapeutic outcomes [205,206,207]. This is evident in vivo*,* where pre-administration of piperine, followed by curcumin orally in rats, resulted in a significant increase in curcumin’s bioavailability after 6 h [208], while in vitro experiments show combined treatment with curcumin and piperine in emulsome nanoformulations induces cell cycle arrest at the G2/M phase and a 6-fold increase in the caspase 3 apoptosis marker in HCT116 colorectal cancer cells [209]. While this herbal combination has not been explored in clinical trials, a case study of a myeloma patient self-administering a daily curcumin supplement complexed with bioperine (a piperine extract) showed sustainable clinical outcomes, including good quality of life, in the absence of antimyeloma treatment and despite approaching a third relapse [210].

Bioavailability and efficacy can also be enhanced by the gut microbiome, which metabolizes certain compounds. For instance, baicalein and baicalin (from *S. baicalensis*) can induce apoptosis in HCT116, SW480, and HT29 CRC cells by modulating the MAPK/ERK and p38 signaling pathways (Figure 2) [211]. While preclinical studies suggest baicalein is more potent than baicalin in these cellular models, its clinical translation is reduced by baicalin’s poor bioavailability (2.2%), attributed to structural differences—presence of a sugar moiety that reduces membrane permeability [211,212]. Notably, intestinal microbiota has been found to metabolize baicalin into baicalein, enhancing its bioavailability and blood concentration [213], a process that is in line with traditional medicine that prescribes oral administration of *S. baicalensis*. This alignment of traditional knowledge and science suggests that there could be a role for the gut microbiome in enhancing therapeutic efficacy. Current research in natural products often adopts reductionist methodologies, focusing on parent compounds and overlooking the metabolites generated by gut microbiota [213]. Thus, cellular models lacking a microbiome step may inadequately represent the biological effects of these constituents and reduce clinical translation. However, incorporating this step as part of the preclinical approaches also has its own challenges.

Preclinical in vivo studies provide insights into the complex interactions between phytoconstituents and cancer pathways, including their safety, efficacy, and potential side effects in living organisms. For instance, oral administration of sanguinarine suppresses tumor growth without observable toxicity in a rat syngeneic model of colorectal cancer [184]. Additionally, sanguinarine regulates the Wnt/β-catenin pathway and reduces tumor angiogenesis in Lewis lung carcinoma models, suggesting potential applications in immunotherapy and antiangiogenic treatments [214]. Similarly, berberine has been shown to suppress tumor growth in human CRC adenocarcinoma xenografts in nude mice by inducing cell cycle arrest and downregulating the expression of related cyclins [215]. It also regulates key pathways, such as the Sin3A/TOP2B pathway in NSCLC xenografts, leading to DNA damage and apoptosis [215,216]. In contrast, curcumin exhibits variable effects across different cancer models in vivo. While curcumin reduces tumor volume in colorectal carcinoma xenografts when delivered via polymeric micelles and intravenous glucuronide formulations [217,218], it paradoxically promotes lesion progression in transgenic lung cancer mouse models, potentially due to pro-oxidant effects in oxygen-rich environments [219]. On the other hand, advances in formulation strategies, such as curcumin-loaded solid lipid nanoparticles (Cur-SLNs), have improved bioavailability and enhanced tumor targeting, significantly increasing curcumin’s efficacy in human lung cancer xenografts [220]. These studies highlight the need for concern when extrapolating findings across cancer types and the need for focused cancer treatment formulations. Although traditional preparations of sanguinarine, berberine, and curcumin remain untested, these in vivo studies provide insights into the efficacy and safety of phytoconstituents while highlighting the challenges of translating traditional medicinal plant research into clinical applications.

Recognizing the complex interactions and potential synergies of phytoconstituents involves exploring the efficacy of whole herb preparations in clinical trials. Currently, herbal preparations of *Zingiber officinale, Astragalus membranaceus, Glycyrrhiza glabra,* and *Codonopsis pilosula* are being evaluated in clinical trials (NCT01344538, NCT03268655, NCT01802021, NCT04690283, NCT00176631, NCT05613465, NCT03616444). These preparations may offer synergistic effects by targeting multiple receptors and pathways, potentially enhancing therapeutic outcomes compared to isolated constituents [221]. The combinations of bioactive constituents in these herbs can provide mechanisms of action, including immunomodulation, reversal of drug resistance, and reduction of adverse effects [222]. This approach aligns with traditional medicine practices which favor whole herbs for their holistic benefits, offering a more comprehensive treatment, targeting multiple symptoms and pathways. This is also starting to be obvious from in vitro studies where various phytochemicals influence the key signaling pathways involved in CRC, such as MAPK, mTOR, PI3K/AKT, and JAK/STAT, with the potential for synergistic effects that could enhance anticancer outcomes [7,9]. For example, in Figure 2, compounds such as lobeline, curcumin, and wogonin target MAPK, mTOR, and PI3K/AKT to reduce proliferation, while curcumin and Astragalus saponins inhibit angiogenesis through the COX-2/VEGF-dependent pathway [94,178]. Additionally, compounds such as glycyrol and baicalein affect the Wnt/β-catenin pathway to reduce cell migration [116,155]. Inflammatory pathways, such as JAK/STAT, MAPK, and NF-κB, not only influence cellular behavior, but are also involved in iron homeostasis, which has been associated with ferroptosis [223]. In this context, compounds such as 18β-glycyrrhetinic acid and wogonin, which block JAK/STAT and reduce migration and invasion, could potentially act in synergy with curcumin, curdione, and glycyrol, which may promote ferroptosis through non-regulated forms of cell death [129,133,146,147,155,157]. Thus, the combination of phytocompounds may offer synergistic potential, enhancing therapeutic efficacy by targeting multiple key cancer pathways, improving outcomes, reducing resistance, and promoting a more comprehensive anticancer effect [224], in line with the holistic approach of traditional medicine, which targets multiple pathways for broader treatment.

Recently, the field of microRNA (miRNA) research has emerged as a promising frontier in cancer biology [225]. These short non-coding RNA molecules, discovered in the early 1990s, stemming from studies on gene regulation in *C. elegans*, have revolutionized the understanding of gene expression control and cancer biology [226]. These molecules play critical roles in cancer pathogenesis, particularly in lung and colorectal cancers, where they influence tumor initiation, progression, and drug resistance by regulating key signaling pathways such as PI3K/AKT and Wnt/β-catenin [94,136,144,162,173]. The rapid advancement in miRNA research has been driven by technological innovations such as high-throughput sequencing, CRISPR/Cas9 gene editing, and advanced imaging techniques [227]. These cutting-edge tools have enabled researchers to study the complex roles of miRNAs in cancer, demonstrating that scientific progress in this field is driven by a combination of curiosity-driven research, technological advancements, and interdisciplinary collaborations [227,228]. While miRNA-based therapies are being explored for various cancers, such as MRG-106 (an oligonucleotide inhibitor of miR-155) for cutaneous T-cell lymphoma and a miR-16 mimic for thoracic cancer [229], their potential in lung and colorectal cancers remains largely unexplored. Promising therapeutic leads may also include phytoconstituents from traditional medicinal plants, which have been shown to modulate miRNA and mRNA pathways (Table 1). For instance, curcumin from *Curcuma longa* reduces the expression of miR-130a, which regulates drug susceptibility through Wnt signaling, while baicalin from *Scutellaria baicalensis* induces apoptosis by inhibiting c-Myc and oncomiRNAs in colorectal cancer. These examples illustrate how the field of miRNA research utilizing modern scientific approaches can still draw from traditional knowledge to develop innovative strategies for cancer treatment.

While cutting-edge research explores miRNA-based therapies, traditional medicinal plants continue to offer valuable insights, as demonstrated by *Althaea officinalis* (marshmallow). It has a rich history in traditional medicine across various cultures, used in lotions and decoctions for treating genital cancers, in traditional Chinese medicine for throat, mucous membrane, and respiratory system illnesses, and prescribed as a hot infusion by herbal medicine practitioners in Lebanon for inflammation, gastritis, and common cold [28,230,231]. Modern research has identified active compounds in *A. officinalis,* such as quercetin, kempferol, and daidzein, which exhibit antiviral properties against cancer-associated viruses, including Kaposi’s sarcoma-associated herpesvirus, Epstein–Barr virus, and hepatitis C virus [181,232,233]. This antiviral activity is particularly significant given that virus-associated cancers account for approximately 20% of the global cancer cases [234]. *A. officinalis* constituents modulate multiple cancer-related pathways, including ERK1/2, PI3K/AKT/mTOR, and apoptosis signaling, reflecting the effects observed in other medicinal plants such as *Zingiber officinale* and *Astragalus membranaceus* (Table 1). While primarily administered for its immune-modulating properties in cancer patients [28,34,235], *A. officinalis* may exert direct anticancer effects on virus-induced malignancies through its antiviral properties. Beyond its antiviral activity*, A. officinalis* extracts have demonstrated promising anticancer effects. Its root extract has been found to enhance cisplatin’s efficacy against A549 lung cancer cells, while its flower extract exhibits antiproliferative effects in lung and colorectal cancer cell lines [180,181]. These outcomes may result from the synergistic action of phenolic acids and flavonoids in the crude extract. However, preclinical mechanistic studies have predominantly focused on individual phytoconstituents, while crude extracts are more commonly used in clinical practice [34,230,231]. This discrepancy highlights the need for an evidence base for clinically used extracts and greater alignment between preclinical testing of herbal preparations and their clinical administration. Further studies are essential to elucidate the cancer signaling pathways regulated by complex herbal extracts, thereby providing a robust evidence-based rationale for their use in traditional and naturopathic practices, as well as informing the development of new therapeutics based on traditional knowledge.

## 6. Limitations

Limitations of this review include focusing on two specific cancers (lung and colorectal) and discussing only the herbs that are commonly utilized across documented traditional medicine and clinical naturopathic practice. This narrative review aimed to provide a contextual understanding of cellular signaling pathways regulated by key phytoconstituents in the herbs discussed based on existing literature. While a systematic review with focused research questions or definitive guideline statements might offer a more comprehensive coverage of all the pathways and constituents present in these herbs, this narrative review serves as a foundational overview within its scope.

## 7. Conclusions

In conclusion, this review summarizes the mechanisms of action of the key phytoconstituents present in various traditional herbal medicines used in the traditional and contemporary management of lung and colorectal cancer. It highlights the modulation of key signaling pathways, such as PI3K/AKT/mTOR, RAS/RAF/MAPK, Wnt/β-catenin, and TGF-β, by isolated compounds such as curcumin, baicalin, berberine, and gingerol, as well as by herbs such as *Echinacea* sp., *Glycyrrhiza* sp., and *Codonopsis* sp., all of which demonstrate significant anticancer properties. By combining the traditional knowledge, contemporary clinical practice, and cutting-edge scientific methodologies, this review draws attention to understanding the impact of the specific herbs and phytoconstituents on signaling pathways—as solo constituents, as a solo herbal extract, and as combined herbal extracts—highlighting the importance of understanding synergy within and between herbs. This has the potential to contribute to a more integrative clinical practice, new anticancer drug development, and a deeper understanding of the impact traditional medicine practices have on contemporary clinical practice across the globe.

## Data Availability

Not applicable.

## Abbreviations

ACSL4—Acyl-CoA Synthetase Long-Chain Family Member 4, AKT—Protein Kinase B, ALOXs—Arachidonate Lipoxygenases, AMPK—AMP-Activated Protein Kinase, APC—Adenomatous Polyposis Coli, APS—Astragalus Polysaccharide, ASCT2—Alanine-Serine-Cysteine Transporter 2, ATF-2—Activating Transcription Factor 2, ATF3—Activating Transcription Factor 3, BAX—Bcl-2-associated X protein, Bcl-2—B-cell Lymphoma 2, BRAF—B-Raf Proto-Oncogene, caspase—Cysteine-Aspartic Protease, CDK—Cyclin-Dependent Kinase, CDK2—Cyclin-Dependent Kinase 2, CDK4—Cyclin-Dependent Kinase 4, c-IAP1—Cellular Inhibitor of Apoptosis Protein 1, c-Myc—Cellular Myelocytomatosis Oncogene, COX-2—Cyclooxygenase-2, CRC—Colorectal Cancer, CREB—cAMP Response Element-Binding Protein, EGFR—Epidermal Growth Factor Receptor, EGR2—Early Growth Response 2, ERK—Extracellular Signal-Regulated Kinase, ERM—Ezrin/Radixin/Moesin, FAK—Focal Adhesion Kinase, FGFR-2—Fibroblast Growth Factor Receptor 2, FZKA—Fuzheng Kangai Decoction, G1—Gap 1 Phase (of the cell cycle), G2/M—Gap 2/mitosis phase (of the cell cycle), GPX4—Glutathione Peroxidase 4, GSH—Glutathione, GSK-3β—Glycogen Synthase Kinase 3 Beta, HIF-1—Hypoxia-Inducible Factor 1, HSP90—Heat Shock Protein 90, IGF1R—Insulin-Like Growth Factor 1 Receptor, IL—Interleukin, JAK—Janus Kinase, JAK/STAT—Janus Kinase/Signal Transducer and Activator of Transcription, JAK2—Janus Kinase 2, JNK—c-Jun N-terminal Kinase, KRAS—Kirsten Rat Sarcoma Viral Oncogene Homolog, LPCAT3—Lysophosphatidylcholine Acyltransferase 3, MAPK—Mitogen-Activated Protein Kinase, Mcl-1—Myeloid Cell Leukemia 1, MDA—Malondialdehyde, MEK—Mitogen-Activated Protein Kinase, miR—MicroRNA, miRNA—MicroRNA, MMP—Matrix Metalloproteinase, MMP-2—Matrix Metalloproteinase 2, mTOR—Mechanistic Target of Rapamycin, NF-kB—Nuclear Factor Kappa-Light-Chain-Enhancer of Activated B Cells, NRF2—Nuclear Factor Erythroid 2-Related Factor 2, NSCLC—Non-Small Cell Lung Cancer, PAI—Plasminogen Activator Inhibitor, PAK4—P21-Activated Kinase 4, PARP—Poly (ADP-ribose) Polymerase, PDL-1—Programmed Cell Death Ligand 1, p-ERK—Phosphorylated ERK, P-gp—P-glycoprotein, PI3K—Phosphoinositide 3-Kinase, PI3K/Akt—Phosphoinositide 3-Kinase/Protein Kinase B, PIK3CA—Phosphatidylinositol-4,5-Bisphosphate 3-Kinase Catalytic Subunit Alpha, PMN—Pre-Metastatic Niche, POR—P450 Oxidoreductase, PPAR—Peroxisome Proliferator-Activated Receptor, PPARγ—Peroxisome Proliferator-Activated Receptor Gamma, PRP—Proliferation-related Protein, PTEN—Phosphatase and Tensin Homolog, PUFA—Polyunsaturated Fatty Acid, QRHXF—Qing-re-huo-xue Formula, Raf—Rapidly Accelerated Fibrosarcoma, Ras—Rat Sarcoma, ROS—Reactive Oxygen Species, RTK—Receptor Tyrosine Kinase, S1PR1—Sphingosine-1-Phosphate Receptor 1, SCC—Squamous Cell Carcinoma, SLC7A11—Solute Carrier Family 7 Member 11, STAT—Signal Transducer and Activator of Transcription, STAT3—Signal Transducer and Activator of Transcription 3, TFEB—Transcription Factor EB, TGF—Transforming Growth Factor, TGFBR2—Transforming Growth Factor Beta Receptor 2, TGF-β—Transforming Growth Factor Beta, THP-1—Human Leukemia Monocytic Cells, TIMP-2—Tissue Inhibitor of Metalloproteinase-2, TNF-α—Tumor Necrosis Factor Alpha, u-PA—Urokinase-Plasminogen Activator, VEGF—Vascular Endothelial Growth Factor, Wnt—Wingless-Related Integration Site, Wnt/β-catenin—Wingless/Int Signaling Pathway, XIAP—X-Linked Inhibitor of Apoptosis Protein, β-catenin—Beta-Catenin.
